# Innovation in immediate neonatal care: development of the Bedside Assessment, Stabilisation and Initial Cardiorespiratory Support (BASICS) trolley

**DOI:** 10.1136/bmjinnov-2014-000017

**Published:** 2015-04

**Authors:** A D Weeks, P Watt, C W Yoxall, A Gallagher, A Burleigh, S Bewley, A M Heuchan, L Duley

**Affiliations:** 1 Department of Women's and Children's Health, University of Liverpool, Liverpool, UK; 2 Department of Clinical Engineering, University of Liverpool, Liverpool, UK; 3 Liverpool Women's NHS Foundation Trust, Liverpool, UK; 4 Worcestershire Royal Hospital, Worcester, UK; 5 St James Hospital, Leeds, UK; 6 Division of Women's Health, Kings College London, London, UK; 7 Royal Hospital for Sick Children and Southern General Maternity Hospital, Glasgow, UK; 8 Nottingham Clinical Trials Unit, University of Nottingham, Nottingham, UK

**Keywords:** Obstetrics, Intensive Care, Delivery

## Abstract

**Objective:**

Babies receive oxygen through their umbilical cord while in the uterus and for a few minutes after birth. Currently, if the baby is not breathing well at birth, the cord is cut so as to transfer the newborn to a resuscitation unit. We sought to develop a mobile resuscitation trolley on which newly born babies can be resuscitated while still receiving oxygenated blood and the ‘placental transfusion’ through the umbilical cord. This would also prevent separation of the mother and baby in the first minutes after birth.

**Design:**

Multidisciplinary iterative product development.

**Setting:**

Clinical Engineering Department of a University Teaching Hospital.

**Methods:**

Following an initial design meeting, a series of prototypes were developed. At each stage, the prototype was reviewed by a team of experts in the laboratory and in the hospital delivery suite to determine ease of use and fitness for purpose. A commercial company was identified to collaborate on the trolley's development and secure marking with the Conformité Européenne mark, allowing the trolley to be introduced into clinical practice.

**Results:**

The trolley is a small mobile resuscitation unit based on the concept of an overbed hospital table. It can be manoeuvred to within 50 cm of the mother's pelvis so that the umbilical cord can remain intact during resuscitation, irrespective of whether the baby is born naturally, by instrumental delivery or by caesarean section. Warmth for the newborn comes from a heated mattress and the trolley has the facility to provide suction, oxygen and air.

**Conclusions:**

This is the first mobile resuscitation device designed specifically to facilitate newborn resuscitation at the bedside and with an intact cord. The next step is to assess its safety, its acceptability to clinicians and parents, and to determine whether it allows resuscitation with an intact cord.

## Introduction

Obstetricians have encouraged early, or even immediate, cord clamping since the 1960s as part of the active management of the third stage of labour to prevent maternal haemorrhage.^1^ For term births, timing of cord clamping does not have a clear effect on postpartum blood loss. However, immediate clamping reduces neonatal haemoglobin and neonatal jaundice.^2^ Although these effects are generally transient and well tolerated, reduced iron levels are still seen at age 3–6 months, an effect associated with developmental delay.^3^ ^4^ The WHO,^5^ the International Federation of Gynaecology and Obstetrics,^6^ and the National Institute for Health and Care Excellence (NICE) in the UK^7^ now recommend deferring cord clamping.

In the UK, about a third of newborn babies are attended at birth by neonatal resuscitation staff. For most, all that happens is an assessment, stimulation, thermal care and simple airway management. Around 15% of babies receive active resuscitation at birth, such as mask ventilation, intubation, cardiac massage or drug administration.

When a baby requires assessment or stabilisation and support at birth, standard practice heretofore is to clamp and cut the cord immediately and then take the baby to a resuscitation platform, usually at the side of the room or in another room. This means that the period of transitional circulation is foreshortened. In addition, parents are often not able to see or touch their baby at birth.^8^ Evidence from other areas of adult and child resuscitation has shown that family presence is preferred by relatives and by staff^9–13^ and is now standard in these settings. This issue has not been explored for care after birth, and to date newborn resuscitation has always been away from the woman and her partner.

The need for immediate neonatal care and support increases with increasing prematurity. For preterm infants, deferred cord clamping is associated with decreased transfusion for anaemia, decreased low blood pressure requiring inotropic support and less low-grade intraventricular haemorrhage compared with immediate clamping.^14^ There is an increase in jaundice, but the long-term effects are unclear. Systematic review suggests that strategies for increased placental transfusion after birth may improve neonatal mortality rate in very premature babies,^15^ but it is uncertain whether any benefits would be negated by delayed resuscitation,^16^ and the current European Resuscitation Council recommendation therefore states that “for babies requiring resuscitation, resuscitative intervention remains the priority.”^17^

Very preterm babies are those most likely to experience these major morbidities and, potentially, might benefit from deferred clamping. It therefore seems important to develop a strategy to allow initial resuscitation with the cord intact. A survey of the UK Extended Neonatal Network in 2009 to assess views on timing of cord clamping and placental transfusion found that two-thirds of those surveyed thought that initial care with the cord intact is potentially feasible for preterm vaginal births. Just under half thought this was possible for preterm caesarean births (Duley, personal communication, 2009).

The aim of this paper is to describe development and preliminary testing of a mobile trolley to enable newborn care and support at the bedside, potentially with an intact cord.

## Methods

### Initial concept

**Figure 1:**
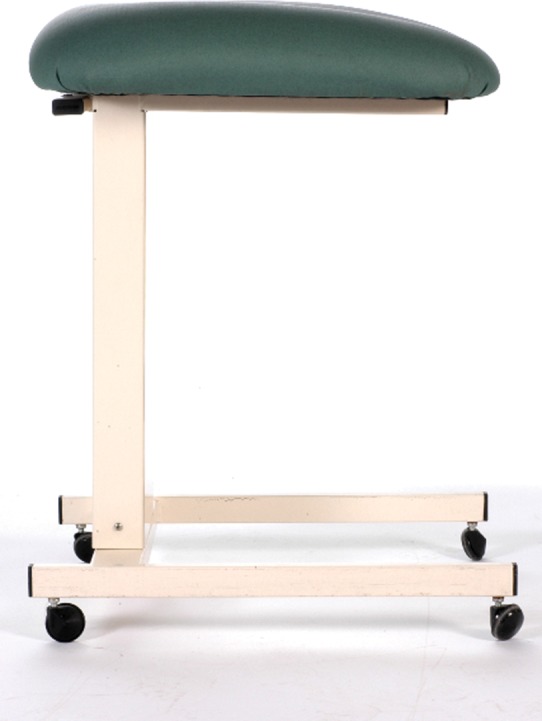
The Glasgow trolley used in the study by Aladangady *et al*^18^ that inspired the first designs of the Bedside Assessment, Stabilisation and Initial Cardiorespiratory Support (BASICS) trolley (photograph courtesy of AMH).

**Figure 2:**
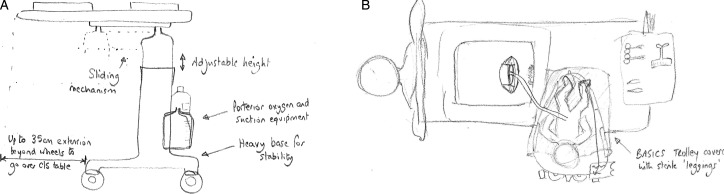
The design drawing that came out of discussions at the first group meeting in Worcestershire showing (A) the trolley design and (B) the way in which it might be positioned over the operating table at caesarean section (drawings in February 2010 by ADW, reprinted with his permission).

Resuscitation with an intact cord has been described by several authors,^18–21^ but no agreed technique had emerged. In January 2010, DH convened a 1-day meeting in Worcester of eight UK clinicians and researchers (AB, SB, LD, AG, AMH, DH, DO and ADW - see Acknowledgement) with a common interest in practical methods for initiating neonatal assessment and resuscitation at birth before the cord is clamped and cut. From this came the idea of a small mobile bedside resuscitation trolley, extending the concept of the platform that had been used for a cord clamping study in Glasgow (figure 1).^18^ The acronym BASICS for ‘Bedside Assessment, Stabilisation and Initial Cardiorespiratory Support’ was proposed, and this established a working title of the ‘BASICS trolley’. The key concepts were that this trolley would need to keep the baby warm, allow suction and respiratory support within a 50 cm radius from the mother's uterus. ADW drew up designs during the meeting (figure 2). It was agreed that he would formalise these, take the design of the trolley forward with the medical engineering department in Liverpool and register the design rights.

### Development of the prototypes

**Figure 3:**
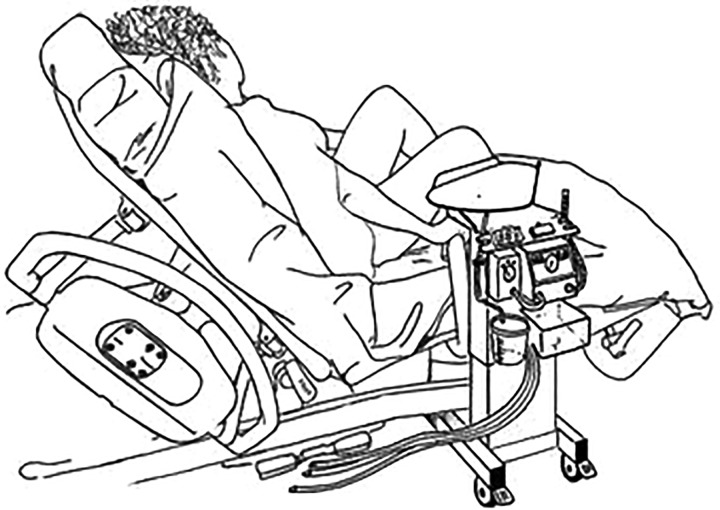
Drawing of the trolley's position for normal delivery (drawing by PW in March 2011, reprinted with his permission).

**Figure 4:**
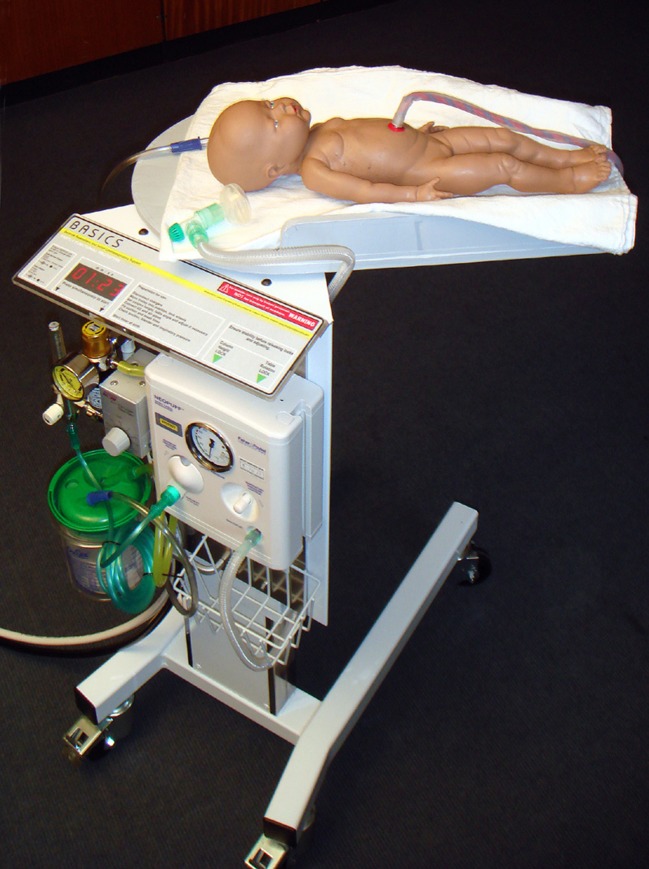
The first Bedside Assessment, Stabilisation and Initial Cardiorespiratory Support (BASICS) prototype (by PW in April 2011, reprinted with his permission). Note that the problem of how to keep the baby warm was still not resolved.

Funding to support development of the concept into a prototype was secured from the National Institute of Health Research (NIHR; LD), with additional support from the Liverpool Women's Hospital ‘Newborn Appeal’. In collaboration with the Department of Clinical Engineering at the Royal Liverpool University Hospital, the first prototype was developed by PW, using a modified hospital overbed table (figure 3). This first early prototype was developed with support from the development team (LD, AG, DH, PW, ADW and CWY), which met in Liverpool, and included input from a service user representative. Various mock delivery scenarios were staged to assess the optimum size and reach of the trolley platform. Discussions included what equipment needed to be available on the trolley to provide blended oxygen and air and to provide positive end-expiratory pressure. The Liverpool prototype (figure 4) was awarded ‘Best Redesign in Cardiovascular Medicine’ at the Medical Futures Awards in June 2011.

**Figure 5:**
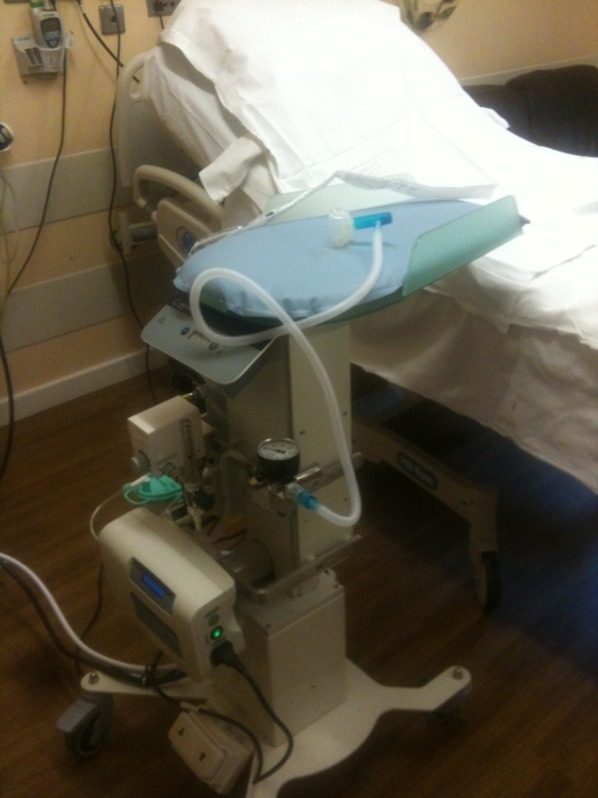
The LifeStart trolley manufactured by Inditherm (October 2012, with permission).

A key problem for the first prototype was how to keep the baby warm. Traditional resuscitaires have an overhead heater, but this was not practical for a trolley designed to go under the legs of a woman in lithotomy position or over an operating table. A commercial company (Inditherm plc) was approached to adapt their heated mattresses for the trolley. The company then agreed to collaborate on further development, with the aim of taking the product to market. The second prototype, developed in collaboration with Inditherm and manufactured by them (figure 5), has improved mobility and flexibility that allows the height and position of the trolley platform to be adjusted. This trolley was marked with the Conformité Européenne (CE) logo in October 2012 and is now marketed by Inditherm as ‘LifeStart’.

### BASICS trolley design

A key element in the design is flexibility to allow the baby to be placed on the trolley while the umbilical cord is still intact. This required the trolley platform to be manoeuvrable: able to be lowered under the mother's legs when in lithotomy position or raised up high to reach over the woman on an operating table. The umbilical cord can be short, so the platform has to be able to be placed close to the woman's introitus. This is achieved by having a central pillar that can be adjusted up or down, and a narrow platform that reaches out from the pillar with raised edges for safety. Even if the mother delivers the baby on the floor, the trolley can be moved to the site of birth and provide the necessary resuscitation gases and suction for the baby to remain attached and with the mother.

Thermal support is provided by the CosyTherm electric heated mattress. This is adjustable through a range of temperatures and takes only a few minutes to heat up. The trolley has a timer built into the platform, with audible Apgar reminder bleeps at 1 and 5 min.

Fixed around the central pillar are two universal Medirails for additional equipment. These allow each hospital to customise the trolley to their specific requirements. Our early assessments used a Tom Thumb Infant Resuscitator (Viamed, Keighley, UK), oxygen blender (Inspiration Health Care Ltd., Leicestershire, UK), a suction bottle driven by the wall-supplied air supply (Oxylitre Ltd., Manchester, UK) and the control unit for the CosyTherm heated mattress (Inditherm, Rotherham, UK). We connect it with hoses to the air and oxygen wall supply. Other equipment that could be added is a light on a flexible stalk, a saturation monitor or a storage basket (for keeping the laryngoscope, hats, saturation monitor).

## Results

The trolley was introduced into clinical service at Liverpool Women's Hospital after CE marking. We describe our early experiences here. More formal evaluation of safety and acceptability to clinicians and to parents has been reported separately.^22^

The first use was for an ex utero intrapartum treatment procedure at caesarean section in October 2012. Feedback from staff and parents was positive. Use was then extended to low-risk deliveries. All parents appeared appreciative of being with their baby during the first moments of life. Clinicians have been slower to accept initial bedside care, and expressed anxieties about the parents being so close to the baby during resuscitation. There have also been practical issues to resolve, particularly in the operating theatre where tubing for air and oxygen can be a trip hazard.

It has also been clear that introducing the trolley for initial care and stabilisation at birth requires teamwork. The equipment must be checked and moved into position ready for the birth. Positioning of the trolley and neonatal team close to the woman needs negotiation with midwifery and obstetric teams. Presently, therefore, the neonatal team are called earlier than for traditional care at the roomside. Once the baby is born, the midwifery and obstetric teams need to understand what is happening during care at the bedside, and will often have a role in supporting the woman and her partner, and explaining to them what is happening.

When using the trolley at a caesarean section, a sterile Mayo tray cover is draped over the platform. The neonatal team member who will provide care at birth scrubs and puts on a sterile gown and gloves so as to prevent contamination of the operating field. Once the baby is on the trolley platform, the sterility rules are relaxed, so that the scrubbed neonatal team member can touch and use non-sterile equipment such as the laryngoscope, timer and hat. It is then important that the sterile field is not re-entered or contaminated. If ongoing resuscitation is required, then, after the cord is cut, the trolley can be moved away from the operating table to allow care to continue without interrupting the surgeon.

## Discussion

### Main findings

We have developed a mobile neonatal resuscitation trolley to allow newborn babies to be assessed and resuscitated alongside their mothers with, or without, an intact cord.

### Strengths and limitations

Clinicians and researchers who wish to implement and evaluate deferred cord clamping for all births have previously faced a problem: How do you achieve this in those babies who require immediate resuscitation? This trolley has demonstrated the feasibility of providing initial neonatal care and stabilisation at the bedside, and to do this while the cord is intact. The trolley now requires evaluation to assess its safety in routine clinical service, and its acceptability to clinicians and parents. Although parents of premature babies describe their first physical contact as very significant, it is frequently delayed until some time after birth on the special care baby unit.^8^ With the BASICS trolley, the mother can touch and speak to her newborn baby while it is undergoing assessment, and our initial experience is that parents value this opportunity.^22^

Introducing neonatal care at the bedside requires a multidisciplinary approach. The attending team need to prepare for, and be confident in, providing neonatal resuscitation under more direct intense parental scrutiny. Everyone present at the birth need to understand their role in supporting this care strategy, and supporting the woman and her partner.

Currently ‘deferred’ cord clamping for very premature babies occurs after 30–45 s, even though placental transfusion is likely to continue for longer. This reflects the desire not to delay initial neonatal care and support. The BASICS trolley should allow evaluation of a more physiological approach to cord clamping for all births, including sick and very premature babies. An ongoing pilot randomised trial compares cord clamping within 20 s with clamping after at least 2 min for births below 32 weeks’ gestation (ISRCTN21456601). In this study, some units are using the BASICS trolley while others are using their normal resuscitaire moved to the bedside.^19^ ^21^ The pilot is to assess feasibility of a large UK randomised trial.

## Conclusion

This is the first mobile resuscitation unit designed specifically to facilitate newborn resuscitation at the bedside, with an intact cord. Further evaluation will assess safety and acceptability, and whether resuscitation with an intact cord to allow longer for the neonatal circulation to be established improves outcome.

## References

[1] Downey CL, Bewley S Historical perspectives on umbilical cord clamping and neonatal transition. J R Soc Med 2012;105:325–9. 10.1258/jrsm.2012.110316 22907549 PMC3423128

[2] McDonald SJ, Middleton P Effect of timing of umbilical cord clamping of term infants on maternal and neonatal outcomes. Cochrane Database Syst Rev 2008;(2):CD004074 10.1002/14651858.CD004074.pub3 18425897

[3] Grantham-McGregor S, Ani C A review of studies on the effect of iron deficiency on cognitive development in children. J Nutr 2001;131:649S–66S. 11160596 10.1093/jn/131.2.649S

[4] Sherriff A, Emond A, Bell JC, et al, ALSPAC Study Team. Should infants be screened for anaemia? A prospective study investigating the relation between haemoglobin at 8, 12, and 18 months and development at 18 months. Arch Dis Child 2001;84:480–5. 10.1136/adc.84.6.480 11369562 PMC1718808

[5] World Health Organization. WHO recommendations for the prevention of postpartum haemorrhage. Geneva, WHO, 2006.

[6] Lalonde A, International Federation of Gynecology and Obstetrics. Prevention and treatment of postpartum hemorrhage in low-resource settings. Int J Gynecol Obstet 2012;117:108–18. 10.1016/j.ijgo.2012.03.001 22502595

[7] National Institute for Health and Care Excellence. Intrapartum care. NICE guideline (CG190), 2014. http://www.nice.org.uk/guidance/cg190 (accessed 11 Feb 2015).

[8] Arnold L, Sawyer A, Rabe H, et al, Very Preterm Birth Qualitative Collaborative Group. Parents’ first moments with their very preterm babies: a qualitative study. BMJ Open 2013;3:e002487. 10.1136/bmjopen-2012-002487 PMC3641451 23550091

[9] Boie ET, Moore GP, Brummett C, et al Do parents want to be present during invasive procedures performed on their children in the emergency department? A survey of 400 parents. Ann Emerg Med 1999;34:70–4. 10.1016/S0196-0644(99)70274-X 10381997

[10] Critchell CD, Marik PE Should family members be present during cardiopulmonary resuscitation? A review of the literature. Am J Hosp Palliat Care 2007;24:311–17. 10.1177/1049909107304554 17895495

[11] Moons P, Norekval TM European nursing organizations stand up for family presence during cardiopulmonary resuscitation: a joint position statement. Prog Cardiovasc Nurs 2008;23: 136–9. 10.1111/j.1751-7117.2008.00004.x 19039895

[12] Resuscitation Council. Should relatives witness resuscitation? UK: Resuscitation Council, 1996.

[13] Robinson SM, Mackenzie-Ross S, Campbell Hewson GL, et al Psychological effect of witnessed resuscitation on bereaved relatives. Lancet 1998;352:614–17. 10.1016/S0140-6736(97)12179-1 9746023

[14] Rabe H, Diaz-Rossello JL, Duley L, et al Effect of timing of umbilical cord clamping and other strategies to influence placental transfusion at preterm birth on maternal and infant outcomes. Cochrane Database Syst Rev 2012;8:CD003248 10.1002/14651858.CD003248.pub3 22895933

[15] Backes CH, Rivera BK, Haque U, et al Placental transfusion strategies in very preterm neonates: a systematic review and meta-analysis. Obstet Gynecol 2014;124:47–56. 10.1097/AOG.0000000000000324 24901269

[16] Resuscitation Council (UK). Newborn life support—resuscitation at birth. 3rd edn 2011.

[17] Richmond S, Wyllie J European Resuscitation Council Guidelines for Resuscitation 2010 Section 7. Resuscitation of babies at birth. Resuscitation 2010;81:1389–99. 10.1016/j.resuscitation.2010.08.018 20956046

[18] Aladangady N, McHugh S, Aitchison TC, et al Infants’ blood volume in a controlled trial of placental transfusion at preterm delivery. Pediatrics 2006;117:93–8. 10.1542/peds.2004-1773 16396865

[19] Hutchon DJ, Thakur I Resuscitate with the placental circulation intact. Arch Dis Child 2008;93:451 10.1136/adc.2008.138206 18426948

[20] van Rheenen PF, Brabin BJ A practical approach to timing cord clamping in resource poor settings. BMJ 2006;333:954–8. 10.1136/bmj.39002.389236.BE 17082547 PMC1633763

[21] Schoonakker B, Dorling J, Oddie S, et al Bedside resuscitation of preterm infants with cord intact is achievable using standard resuscitaire. 54th meeting of the European Society for Pediatric Research; 2013:430 http://www.eiseverywhere.com/file_uploads/9206db9fe962868d47f709b38365ec5e_9349_abstract_book_-_25sett13-it-it.pdf (accessed 13 Feb 2015).

[22] Thomas MR, Yoxall CW, Weeks AD, et al Providing newborn resuscitation at the mother's bedside: assessing the safety, usability and acceptability of a mobile trolley. BMC Pediatr 2014;14:135 10.1186/1471-2431-14-135 24885712 PMC4055396

